# Depression in elderly women resident in a long-stay nursing home

**DOI:** 10.1590/S1980-57642015DN91000012

**Published:** 2015

**Authors:** Melissa Agostini Lampert, Ana Luiza Pereira Rosso

**Affiliations:** 1 Geriatric Medical Doctor at the Hospital Universitário de Santa Maria (HUSM).; 2 Medical Doctor graduated at the Universidade de Santa Maria (June, 2014), Lar das Vovozinhas Long-stay Nursing Home, Santa Maria, RS, Brazil.

**Keywords:** long-stay nursing home, depression in elders, psychiatric disorder

## Abstract

Depression is the most common psychiatric disorder among the elderly: it is present in 23-40% of community-dwelling elderly and in 25-80% of institutionalized elderly. Depressive symptoms are most prevalent in elderly women because they more readily seek healthcare services, are more vulnerable to stress and live longer than men.

**Objective:**

To investigate the prevalence of depression and its comorbidities in a long-stay nursing home (NH).

**Methods:**

This retrospective, descriptive, epidemiological study was performed at a NH in southern Brazil and comprised the first part of a larger project to determine depression and its relationship with psychosocial factors in NH residents. Sociodemographic and medical data were obtained through the examination of medical files from November 2012 to January 2013. Depression was defined as the diagnosis reported by physicians in medical files and scores on the Geriatric Depression Scale (15-item version) above 5. Other clinical and psychiatric diagnoses were obtained from medical files.

**Results:**

Out of a total of 142 elderly women, 51.4% had at least one psychiatric disorder, the most common being depression, affective bipolar disorder and mental retardation. Almost one third (32.3%) of the elderly women were depressed. The ward containing the highest number of cognitively and physically independent women contained 41.3% of the total depressed elderly. A total of 52.1% of all depressed elderly had other associated clinical or psychiatric disorders, with mental retardation and hypothyroidism being the most frequent.

**Conclusion:**

The prevalence of dementia was high in this NH. Further studies evaluating the psychosocial factors involved in depressed elders should be conducted in an effort to prevent depression and promote mental health in institutionalized elders.

## Abstract (pt)

A depressão é a doença psiquiátrica mais comum entre idosos: 23% a 40% da população idosa na comunidade tem esse diagnóstico e entre institucionalizados são 54%. Os sintomas depressivos prevalecem mais em mulheres pois elas procuram mais por serviços de saúde, são mais vulneráveis ao stress e vivem mais que os homens.

**Objetivo:**

Investigar a prevalência de depressão e suas comorbidades em uma Instituição de Longa Permanência (ILP).

**Métodos:**

Este é um estudo retrospectivo descritivo sendo uma análise epidemiológica da incidência de depressão e do perfil de idosas institucionalizadas residindo na ILP Lar das Vovozinhas em Santa Maria RS. Compõe a primeira etapa de um projeto que pretende verificar a presença de depressão em idosas de uma ILP e sua relação com fatores psicossociais. A coleta de dados foi realizada em revisão de prontuários de novembro de 2012 a janeiro de 2013.

**Resultados:**

De 142 idosas da ILP 51,4% possuem algum distúrbio psiquiátrico associado sendo a depressão, o Transtorno Afetivo Bipolar e o retardo mental os mais comuns. 32,3% das idosas apresentam o diagnóstico de depressão. A ala com o maior número de idosas independentes cognitiva e fisicamente possui 41,3% do total de idosas deprimidas. Das idosas deprimidas 52,1% possuem outro distúrbio psiquiátrico ou orgânico associado sendo o retardo mental e o hipotireoidismo os mais presentes.

**Conclusão:**

A prevalência de demência encontrada nessa ILP foi alta. Estudos futuros são necessários para avaliar os fatores psicossociais envolvidos na depressão em idosas visando à prevenção dessa doença e a promoção de saúde mental nessa população.

## INTRODUCTION

Depression is the most common psychiatric disorder in elders, significantly impacting their quality of life.^1^ An estimated 23-40% of the general elderly population has this diagnosis while the rate in institutionalized elderly averages 54%,^2^ but can ranges from 25-80%.^3^ In a previous survey, it was concluded that the majority of institutionalized elders (75%) were dissatisfied with their situation in the institution, felt not enough attention was given to their well-being and had low self-esteem.^4^

Depression in elders has multifactorial origins that can encompass organic factors (e.g.: diseases such as hypothyroidism, stroke, diabetes) and psychosocial factors (e.g.: retirement, mourning, institutionalization).^5^ An estimated 15% of elderly people in Brazil present depressive symptomology associated or otherwise with psychiatric disorders.^6^

There is often an association with physic and mental illness when, in many cases, an underlying organic disease increases the risk for future development of psychiatric disorders.^7^ Illnesses which can predispose toward depression include visual disability, auditory disorder,^8^ Alzheimer's,^9^ COPD, cancer^10^ and also other neurological diseases such as Parkinson's and other dementias.^7^ As depression is a prevalent psychiatric disorder in elders, clinical diseases may contribute to the development or onset of depression by directly affecting brain function or exerting psychological and psychosocial effects.^11^

The disorder among institutionalized elders is of greater concern. Depression is severe in 15-19% cases and mild in at least 50%.^12^ Consequently, many surveys have sought to perform analysis on depression incidence and social profile in elders of long-stay nursing homes (NH).^13,14^

The symptomatology of depressed elders often differs from that of adults with the same pathology. Elders tend to talk a lot generally but little about their feelings, while somatic symptoms can mask depressive symptoms.^15^ Thus, changes such as fatigue, energy loss, as well as sleeping and eating disturbances, can be more evident in depressed elderly than in adult subjects and in many cases are more evident than the emotional symptomatology of depressed patients.^16^

Depression diagnosis is clinical. Besides symptomatology, medical services also employ the Geriatric Depression Scale (GDS).^17^ This scale can be used as a screening instrument for depressed patients or to supplement the diagnosis together with other clinical data (anamnesis, clinical examination and possible complementary exams). The GDS consists of a questionnaire with questions applied by an interviewer in which mood aspects are evaluated. One of the most widely used GDS is the 15-item version, on which scores of over 5 indicate depression.^18^ A number of international surveys have validated the GDS in many different sectors to aid the tracking and diagnosis of depression in elderly people.^19^

Depression has a spectrum of severity ranging from mild to severe, such as suicide. Suicide is a risk among depressives and its rates increase with age and must therefore be considered when dealing with the elderly population.^20^

Depressive symptoms have a higher prevalence in women due to several factors such as more readily seeking health services, higher vulnerability to stressful events,^21^ higher propensity for widowhood and the fact that women live longer than men. The presence of depression is often found to be related to factors concerning the environment in which the elder resides.^22^ Questions have been raised regarding the peculiarities observed in those elderly women living in NHs diagnosed with depression, and this provides the basis for the objective of the present survey.

The aim of the present survey was to determine the incidence of depression in institutionalized elderly women and the epidemiological factors associated with the disorder. The study also sought to identify the association between the environment in which these patients live and occurrence of the disorder.

## METHODS

A descriptive and retrospective survey was carried out with the study factor defined as elderly women with depression living in a long-stay nursing home (NH) and the population studied as elderly women living in the NH "Lar das Vovozinhas".

"Lar das Vovozinhas", situated in the city of Santa Maria - RS, is the biggest NH in Rio Grande do Sul state with a capacity for up to 210 people. It is a philanthropic institution and supported with funds raised through the annual party, community donations, retirement money, contributions by family members and a small public contribution. It takes in elderly women independently of their health situation, as well as younger women with special needs, in socially vulnerable situations with no means of supporting themselves or of being supported by their family.

The present study is part of the project: "Cuidado ao Doente Crônico (Care for the Chronically Ill): A atuação interdisciplinar como espaço potencializador de transformação" CAAE 00690243000-11. The study had the approval of the Institutional Research Board CEP-UFSM (Comitê de Ética em Pesquisa da Universidade Federal de Santa Maria).

The first step was sample definition which took place during October 2012 entailing analysis of the institution's data files holding information about elders living in the NH. The variables gender and age were analyzed and out of a total of 185 people, 43 subjects under 60 years old were excluded, giving a final total of 142 elderly women.

The second step, accomplished from November 2012 to January 2013, involved the analysis of elderly women's medical files observing the follow variables:

- Age: considering the register in the medical file or administrative documentation, as birth date.
- Environment: the place in the NH where the elder stays most of their time was considered and those characteristics having a potential association with the presence of depression were analyzed.
- Presence of depression: a diagnosis of depression was defined when depression was reported by the physician in the medical file according to the clinical symptomatology and when scores on the Geriatric Depression Scale (15-item version) applied by the physician were over 5.
- Presence of other neuropsychiatric disorders: bipolar affective disorder, schizophrenia, mental retardation, psychosis, epilepsy, anxiety and other psychiatric disorder. The presence of psychiatric pathology diagnosis in medical anamnesis or of positive psychiatric reports of the diagnosis defined these disorders.
- Presence of organic pathologies: a group of organic diseases or conditions were selected to check their possible association with depression: hypothyroidism, stroke, communication disorder, Parkinson's disease, dementia, extrapyramidal syndrome and cognitive disorder. Diagnoses were established based on the presence of positive psychiatric or clinical evaluations for the condition in question.

The third step consisted of data compilation and descriptive statistical analysis based on frequency, mean, standard deviation and Student's t-test when necessary. The statistics program SPSS 13.0 was used for all statistical analyses.

## RESULTS

The average age of the 142 elderly women was 74 years (SD:9.365).

Regarding the environment, the physical infrastructure of the NH consists of 4 Wards, each with a specific purpose: Wards 1 and 4: active women with no intensive care necessary; Ward 2: women with mobility restriction; Ward 3: women with a range of care needs associated with behavioral changes and need for vigilance.

The activities performed by elders are divided by Ward according to the degree of available dependence. Ward 1 accommodates semi-independent elderly women many of whom use a wheelchair or walker. It has a living room, activity room and also a bandstand where the elders hold weekly traditional "chimarrão" get-togethers. The residents also pray and go to the church.

Ward 4 has a dining room, living room and handicraft room. Most elders are semi-independent and live together in those places. Some of them also go to church or festive events.

Ward 3 has a large enclosed backyard with nursery for birds, living pool with fish, garden and grotto. Since this ward has many elders with behavioral changes, the social interaction is more restricted and likewise for Ward 2, which accommodates totally dependent elderly women.

Regarding psychiatric pathologies, the distribution of psychiatric pathologies in all elders of the NH and in depressed elderly women only can be seen in Table 1. Similarly, the presence of organic pathologies in these groups may be seen in Table 2. It was concluded that 51.4% of all elders in the NH had at least one diagnosed psychiatric disorder, with depression (32.3%), affective bipolar disorder and mental retardation (9.15% each) being the 3 most frequent.

**Table 1:** Distribution of psychiatric pathologies.

Psychiatric pathologies	All elders from NH (n=142)	%	Depressed elderly (n=46)	%
Bipolar affective disorder	13	9.15%	2	4.34%
Mental retardation	13	9.15%	5	10.58%
Schizophrenia	6	4.22%	1	217%
Other psychiatric disorder	12	8.45%	3	4.34%
Psychosis	6	4.22%	2	6.52%
Epilepsy	4	2.81%	2	6.52%
Total	54	38%	15	34.47%

**Table 2:** Distribution of organic pathologies.

Organic pathologies	All elders from NH (n=142)	%	Depressed elderly (n=46)	%
Hypothyroidism	21	14.7%	6	13.04%
Stroke	12	8.45%	4	8.69%
Communication disorder	9	6.33%	2	4.34%
Dementia	14	9.85%	2	4.34%
Parkinson’s disease	7	4.92%	1	2.17%
Extrapyramidal syndrome	1	0.70%	1	2.17%
Cognitive Disorder	6	4.22%	1	2.17%
Total	70	49.17%	17	36.92%

**Figure 1:**
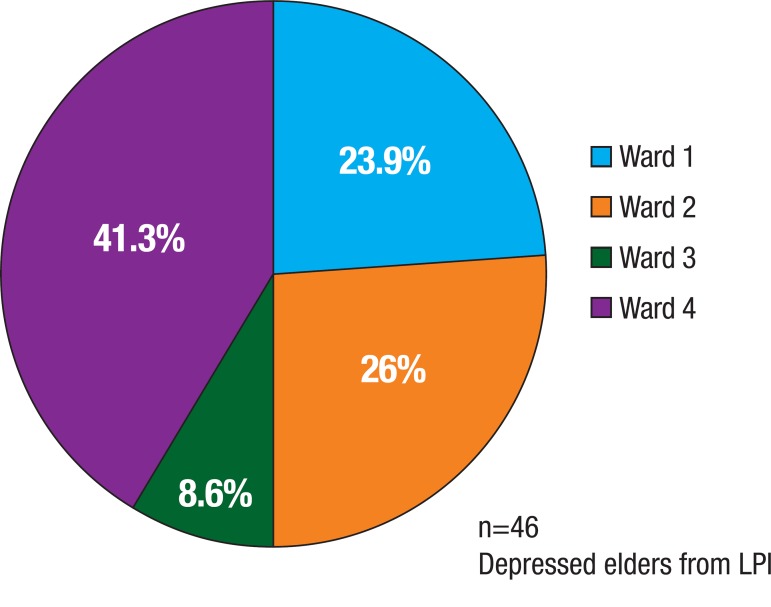
Depressed elders by ward.

Regarding the presence of depression, 32.3% of elders were diagnosed with the condition (n=46). The average age of depressives was 75,3 years (SD:9.37) whereas for non-depressive elderly women was 73.5 years (SD:9.43), not statistically significant on Student's t-test (T<t* ; 95%CI). The ward with the highest number of depressed women was Ward 4 with 19 depressive elders, followed by Ward 2 (12 elders), Ward 1 (11 elders) and lastly Ward 3 (4 elders). The percentage distribution across the Wards can be seen in Figure 1.

Of depressed elders, 52.1% had another psychiatric or organic disorder associated with depression, with hypothyroidism (13.04%) and mental retardation (10.8%) being the most common. The 3 psychiatric pathologies most frequently associated with depression were mental retardation (10.8%) and other psychiatric disorders and epilepsy (6.52% each), followed by affective bipolar disorder, and other psychiatric disorder (4.34% each). The 3 organic pathologies most associated with depression were hypothyroidism (13.04%), stroke(8,6%) and communication disorder (4.34%).

## DISCUSSION

The prevalence rate of depression in institutionalized elderly women in this study was 32%, which is similar to the rates reported in the literature (25-80%).^8^ Notably in the present study, a large number of elderly women had associated psychiatric disorders (51.4%) relative to other studies showing that 30-80% of NH residents had some degree of dementia and 40-90% of demented NH residents were diagnosed with depression, psychosis, aggressiveness or *delirium*.^23^ According to a number of Brazilian studies, the rate of psychiatric disease among elderly in NH ranges from 30.1-80.1%.^24^

The average age found of 74 years old for general elders of the NH is in accordance with other studies. In a study conducted in Juiz de For a, Minas Gerais state, the average age found for the female population was 77.27 years old (SD 8.69).^9^ The association between depression and comorbidity is important because it is frequent in chronically-ill elders: among those who have health problems, depression incidence is 30-70%.^5^ The literature reports an higher incidence of depression in Parkinson's disease (40-60%), Alzheimer's disease (30-40%), stroke (30-60%) and epilepsy (10-50%).^25^ There are also reports of increased depression in elderly with hypothyroidism.^26,27^

Out of all elders with depression, 47.8% had a depression diagnosis with no other associated pathologies (total of 22 elderlies). Other epidemiological variations such as race, years of schooling, marital status and reason for institutionalization, found in other studies were not assessed in the current survey because this information was lacking in the medical files. These data may be considered in subsequent studies observing variations in the social environment and psychological issues that may influence the development of depression in elderly women.

Regarding environment factors, most depressive elders were in Ward 4, accommodating individuals independent for cognitive or physic aspects. This result was somewhat unexpected considering that global health status of these elders was better than for other Wards, and therefore less conducive for the development of depression. However, this finding may be explained by the fact these elders were better able to explain and talk about how they felt.

The results found in this study were based on general information from medical evaluations made by the general physician of the "Lar das Vovozinhas" Nursing Home and were based on data for the GDS 15 and depression symptomatology. These sources represent limitations of the present survey. It is important to bear in mind that this represents the reality of many NHs in Brazil, particularly those maintained by public or charity donations, where not all the necessary medical resources to assist the elderly residents in these institutions are available. Moreover, this survey was performed in only one NH, a philanthropic institution which assists elderly women. If the investigation were to encompass other settings, the results may be different. Therefore, it is important to conduct further studies that reflect the reality of other NHs.

In the present survey, it was concluded that 32.3% of the elderly residents in the NH Lar das Vovozinhas had depression and 52.1% had some any other associated psychiatric or organic condition that may have been related to depression, with mental retardation and hypothyroidism being the most common. Some 47.8% had depression as the only pathology. It can also be concluded that 51.4% of all the elderly had at least one associated psychiatric disorder. The ward with the highest number of depressive elders was Ward 4, which accommodates mostly independent elders. Thus, it was concluded that the environmental factor studied in this survey (the ward in which elders spent most of their time) was insufficient to confirm the association between depression and environment. Possibly, the activities and organization of the wards is insufficient for the prevention and promotion of mental health. More adequate ways of evaluating elders with communication difficulties in NHs should be developed, and other environmental and social factors that may be associated with depression should also be explored. Indeed, this will be the primary objective of the second stage of the survey: to detect the psychosocial aspects influencing the development of depression in these elders. This knowledge will allow the prevention and promotion of mental health among institutionalized elders and enhancement of their quality of life.
